# Thyroid Stimulating Hormone as a Possible Additional COVID-19 Outcome Marker

**DOI:** 10.3390/medicina60020314

**Published:** 2024-02-12

**Authors:** Anamarija Zrilic Vrkljan, Ana Majic Tengg, Tanja Palaversa, Srecko Marusic, Lana Ruzic, Ines Bilic-Curcic, Maja Cigrovski Berkovic

**Affiliations:** 1 Department of Endocrinology, Diabetes, Metabolism and Clinical Pharmacology, Clinical Hospital Dubrava, 10000 Zagreb, Croatia; anamarija.zrilic@gmail.com (A.Z.V.); ana.majic89@gmail.com (A.M.T.); marusic.srecko@gmail.com (S.M.); 2 Levanger Hospital, 7600 Levanger, Norway; tanjarezic1@gmail.com; 3 Department of Exercise and Sport Medicine, University of Zagreb Faculty of Kinesiology, 10000 Zagreb, Croatia; lana.ruzic.svegl@kif.unizg.hr (L.R.); maja.cigrovskiberkovic@gmail.com (M.C.B.); 4 Department of Endocrinology and Diabetes, University Hospital Centre Osijek, 31000 Osijek, Croatia; 5 Faculty of Medicine Osijek, Josip Juraj Strossmayer University of Osijek, 31000 Osijek, Croatia

**Keywords:** SARS-CoV-2, COVID-19, TSH, outcomes

## Abstract

*Background and Objectives*: The interaction between thyroid and SARS-CoV-2 is complex and not yet fully understood. This study aimed to identify a predictive value of serum TSH levels on the short-term and middle-term outcomes of patients hospitalized for COVID-19. *Materials and Methods*: We retrospectively analyzed electronic records (ERs) data for hospitalized COVID-19 patients between March 2020 and June 2021 and their ERs during outpatient visits, 6–8 weeks post-discharge, in cases of known serum TSH levels and no previous thyroid disorder. The short-term (length of hospital stay, MSCT findings of lung involvement, required level of oxygen supplementation, admission to the ICU, and death) and middle-term outcomes after 6 to 8 weeks post-discharge (MSCT findings of lung involvement) were analyzed. *Results*: There were 580 patients included: 302 males and 278 females, average age of 66.39 ± 13.31 years, with no known thyroid disease (TSH mean 1.16 ± 1.8; median 0.80; no value higher than 6.0 mIU/L were included). Higher TSH was observed in patients with less severe outcomes and was associated with significantly higher SpO_2_ during hospitalization. Patients who required overall more oxygen supplementation or HFOT, mechanical ventilation, and patients who were more frequently admitted to the ICU or were more often treated with corticosteroids had lower TSH than those who did not show these indicators of disease severity. Lower TSH was also present in non-survivors when compared to survivors (all *p* < 0.01). Patients with low TSH during hospitalization more often had persistent lung involvement during the post-COVID-19 period (*p* = 0.028). In the post-COVID-19 period, there was an overall, statistically significant increase in the TSH levels when compared to TSH during hospitalization (*p* < 0.001). *Conclusions*: Low/suppressed serum TSH levels during acute COVID-19 may be an additional laboratory test that should be included in the prediction of unfavorable short- and middle-term outcomes.

## 1. Introduction

The coronavirus disease 2019 (COVID-19) pandemic, caused by the severe acute respiratory syndrome coronavirus-2 (SARS-CoV-2), continues to pose a threat to global health. According to the World Health Organization, until December 2023, there were 772,838,745 confirmed cases of COVID-19 worldwide with 6,988,679 confirmed deaths [1]. 

The pathogenesis of COVID-19 involves SARS-CoV-2 entering the respiratory system. When the angiotensin-converting enzyme 2 (ACE2) on the surface of pneumocytes binds to the spike protein of the virus, it facilitates the entry of the virus into cells [2,3]. Numerous organs, including endocrine glands, express ACE2, potentially leading to the extrapulmonary and multiorgan spread of SARS-CoV-2 [4,5]. 

In March 2020, the European Society of Endocrinology issued a statement to raise awareness within the entire endocrine community regarding the role and responsibilities of endocrinologists worldwide during the ongoing COVID-19 outbreak. Individuals with diabetes were identified among those at high risk for serious illness, along with people with obesity, malnutrition, and adrenal insufficiency [6]. An update in April 2021 described an emerging endocrine phenotype of COVID-19, emphasizing the significance of thyroid aspects in the context of COVID-19 [7]. 

It is crucial to gain a deeper understanding of the pathophysiology of the disease and promptly identify the phenotypes of vulnerable patients who are at the highest risk of immediate and prolonged unfavorable outcomes. 

Our study aims to investigate the predictive value of serum Thyroid Stimulating Hormone (TSH) in patients with no known thyroid disorder concerning short-term and middle-term COVID-19 outcomes.

## 2. Materials and Methods

This study was approved by the Ethics Committee of Clinical Hospital Dubrava (approval number 2021/1312-02). Due to the retrospective nature of the study, informed consents were waived. 

### 2.1. Study Participants and Data

We retrospectively analyzed the ER data of patients admitted for COVID-19 to the Clinical Hospital Dubrava in Zagreb, Croatia, between March 2020 and June 2021. The study included patients with no prior history of thyroid disorders and those for whom TSH serum levels were available during hospitalization and in the subsequent post-COVID-19 outpatient clinic visit, 6 to 8 weeks post hospital discharge. Since the onset of the COVID-19 pandemic, Clinical Hospital Dubrava has been designated as a national COVID-19 center, with specialists from various medical fields actively involved in patient care. To streamline processes, predefined checklists for various medical conditions, including thyroid disorders, were implemented. TSH evaluation was not a routine part of the laboratory assessments during hospitalization, but its screening was determined based on the discretion and recommendation of the attending healthcare provider. Consequently, TSH results were available for 580 patients. The TSH level was measured utilizing a chemiluminescent immunoassay method on a Beckman Coulter analyzer type DxI 800. The reference range was 0.38–5.33 mIU/L. For those 580 patients, we assessed short-term outcomes (such as the length of hospital stay, lung involvement measured by Multi-Slice Computed Tomography (MSCT) in 196 patients, required level of oxygen supplementation, admission to the intensive care unit (ICU), and mortality). We excluded patients with known thyroid disease or extreme TSH values (TSH mean 1.16 ± 1.8; median 0.80; no value higher than 6.0 mIU/L). Middle-term outcomes, specifically lung involvement measured by MSCT during the post-COVID-19 period, were examined 6 to 8 weeks after hospital discharge in 147 patients. The diagnosis of COVID-19 was confirmed through real-time reverse transcriptase polymerase chain reaction (RT-PCR) testing of nasopharyngeal swab samples. Within 48 h of admission, patients underwent a standard set of blood tests, including measurements of C-reactive protein (CRP), along with additional tests, such as interleukin-6 (IL-6), procalcitonin (PCT), and D-dimer, the timing of which relied upon clinical indication. Laboratory analyses were performed at the Clinical Department of Laboratory Diagnostics, an ISO 15189:2022 [8] accredited laboratory, within Clinical Hospital Dubrava. Parameters for assessing the severity of COVID-19 infection were documented during hospitalization and included the duration of hospital stay, the level of required oxygen supplementation, admission to the ICU, and mortality rates. These parameters were utilized to estimate short-term outcomes associated with COVID-19. Both short- and middle-term complications of COVID-19 were assessed using chest MSCT images, when available during hospitalization and in the post-COVID-19 outpatient clinic. Additionally, TSH levels were again considered during the outpatient clinic visit. The percentage of lungs affected by inflammatory changes related to COVID-19 infection was evaluated on MSCT and included “ground glass” opacification, consolidation, bronchiectasis/bronchiolectasis, reticulation, parenchymal bands, and honeycombing, as defined in the Fleischner Society glossary [9]. Patients were categorized into groups based on the percentage of pulmonary involvement: Group 1—up to 30% of pulmonary parenchymal involvement; Group 2—more than 30% of pulmonary parenchymal involvement. Chest MSCT was performed for 196 patients during hospitalization based on indications such as suspicion of pulmonary embolism, prolonged need for oxygen supplementation, or worsening of the patient’s condition. In the post-COVID-19 outpatient clinic, chest MSCT was performed for all patients.

### 2.2. Statistical Methods

The data were analyzed using Statistica (TIBCO) software version 14.0.0.15. Descriptive statistics were employed to outline the basic features of the study sample. The normality of distribution was assessed using the Shapiro–Wilk test but distributions of different groups according to outcome were also analyzed and inspected through variability plots. Mann–Whitney U test, Wilcoxon matched pairs, or parametric dependent and independent Student *t*-test was used when comparing two groups or changes in one group and we also opted for parametric ANOVA as a robust method when comparing more groups after inspecting differences in variability plots among groups. 

## 3. Results

Serum TSH levels were available for 580 hospitalized COVID-19 patients with no previously known thyroid disorders, and these patients were included in our study. Not all the patients included had all variables of interest measured, so the numbers of patients for each parameter are shown in Table 1. The study cohort comprised 302 male and 278 female patients, with a mean age of 66.39 ± 13.31 years. Additional details regarding patients’ characteristics and their hospital stay and laboratory findings are presented in Table 1. 

### 3.1. Short-Term Outcomes and TSH 

**Figure 1:**
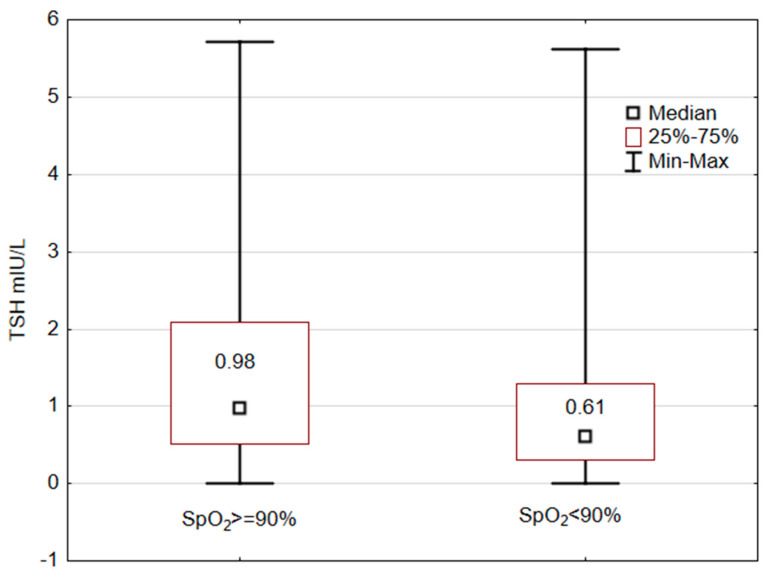
Peripheral blood oxygen saturation and TSH levels.

The sample was first divided according to the median of peripheral blood oxygen saturation. The group with lower SpO_2_ (lower than 90% had significantly lower average TSH values (Mann–Whitney U test: U = 27792.5, Z = 4.958; *p* < 0.001) than the group with better SpO_2_ (Figure 1). 

Higher TSH levels were seen among the COVID-19 survivors (out of all 580 analyzed cases 161 patients died; 26% males and 29.5% females). On the other hand, patients with lower TSH levels were more frequently transferred to the intensive care unit (ICU), required mechanical ventilation, or died (all *p* < 0.01), Table 2. 

**Figure 2:**
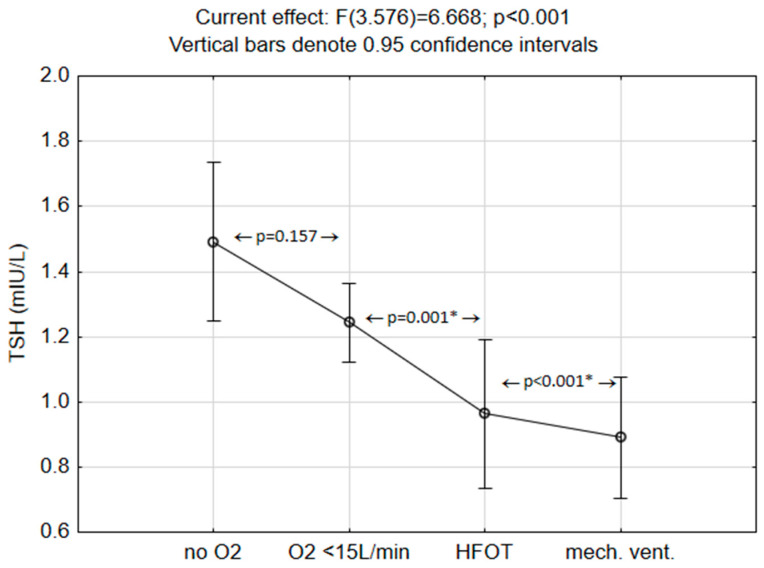
Differences in TSH according to the oxygen demand during hospitalization. * Asterix represent significant differences in post hoc test: no significant difference was found between the group that did not need oxygen and the group who needed up to 15 LO_2_/min, while the groups that needed high flow oxygen (HFOT) or mechanical ventilation differed significantly from each other as well as from the first two groups (NoO_2_− patients with no need for oxygen; O_2_ < 15 L/min—patients who needed O_2_ but less than 15 L/min; HFOT—patients on high flow oxygen therapy; mech.vent—patients who needed mechanical ventilation).

Moreover, patients were divided into four groups according to oxygen demand during hospitalization, and TSH levels during hospitalization were analyzed for each group. The demand for oxygen supplementation rose significantly with the lowering/suppression of TSH. The difference was not observed between those requiring no oxygen and those who needed up to 15 L/min of oxygen, while all other groups differed significantly. Even though the Kruskall Wallis test showed a significant difference between the four groups (Chi = 14.37; *p* = 0.002), ANOVA was used after inspecting the variability plots for their robustness against slight normality violation and the results are depicted in Figure 2.

A small but significant negative correlation between the highest oxygen flow in liters and TSH during hospitalization was also observed (Spearman R = −0.30), again confirming higher TSH in patients with lower oxygen demand. 

No significant correlations were noted between the TSH level and age of the patients (Spearman R −0.013), BMI (Spearman R −0.060), D-dimer concentration (Spearman R −0.036), or IL-6 (Spearman R −0.030). On the other hand, there was a very weak but significant correlation between TSH level and procalcitonin (Spearman R −0.107), and TSH and CRP (Spearman R −0.143).

The majority of our patients required corticosteroid therapy, i.e., only 12% of male patients and 13% of female patients were not administered corticosteroids. 

Significantly lower TSH levels were seen in female patients receiving corticosteroids than in those who received no such therapy (median 0.74 vs. 1.14 mIU/L; *p* < 0.01), while there was no statistical difference in TSH concerning need for corticosteroids in men, even though the medians and rank sums also differed in favor of the no corticosteroids group (median 0.73 vs. 1.02 mIU/L; *p* = 0.435), Table 3.

**Figure 3:**
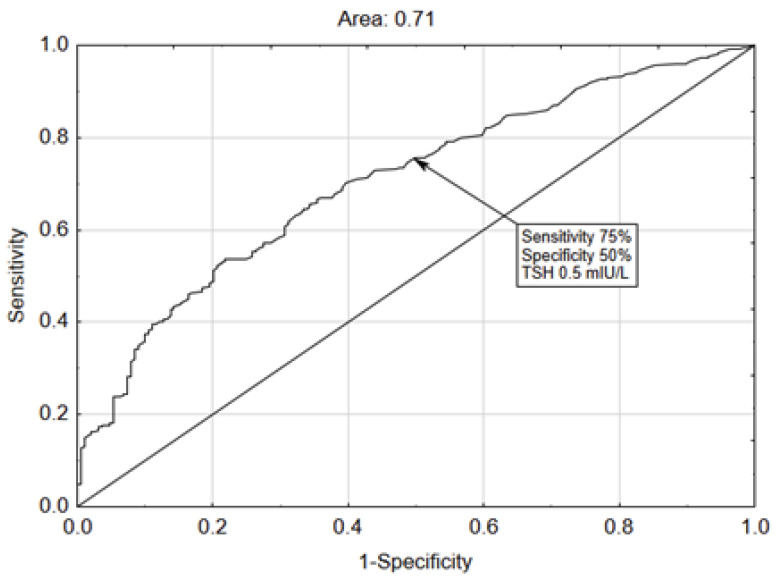
ROC analysis curve with an AUC of 0.71. The selected cut-off point compromises between sensitivity and specificity, favoring sensitivity.

The total number of patients who underwent MSCT during hospitalization was 196. All the patients who underwent MSCT had at least some lung tissue involvement. The distribution of the patients according to lung involvement is shown in Figure 3. Men and women were equally affected. No relation was found between the lung involvement during hospitalization and TSH during hospitalization.

### 3.2. TSH as a Predictor of Worse Outcome

An ROC analysis was conducted to assess the possible predictive value of TSH in determining the need for mechanical ventilation. The AUC of 0.705 (95% CI 0.662–0.749) was indicative of only a fair to moderate level of discrimination between the patients who needed mechanical ventilation and those who did not (Figure 3). The cut-off point determined from the curve which might serve as an additional aid for a clinician to prepare for a worse outcome was 0.5 mIU/L (the cut-off point was set towards higher sensitivity of 75% and less specificity, only 50%).

### 3.3. Middle-Term Outcomes and TSH

TSH was analyzed in 207 patients during post-COVID-19 follow-up. In the post-COVID-19 period, there was an overall increase in TSH levels compared to those measured during hospitalization (Wilcoxon’s test for paired samples *p* < 0.001), Table 4.

**Figure 4:**
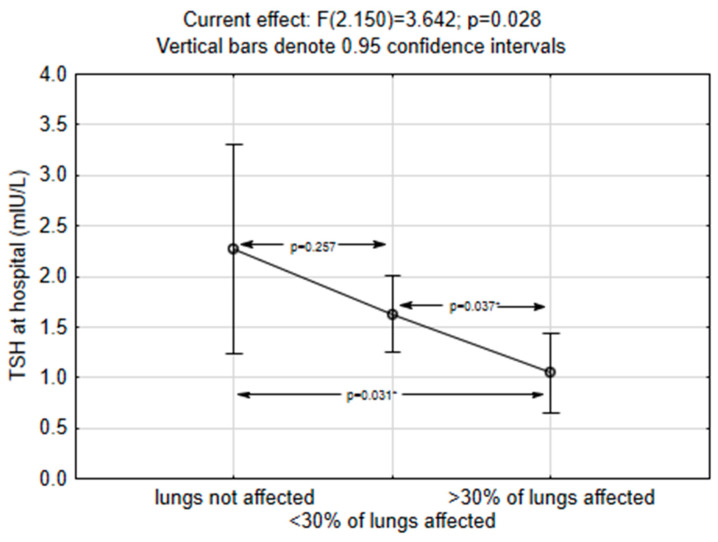
Persistence of lung damage in the post-COVID-19 according to TSH levels (* Asterix represents *p* values with significant differences between the groups without lung tissue involvement and with more than 30% of lung tissue affected (*p* = 0.031), as well as between the group with less than 30% of lung tissue involvement and the group with more than 30% of lungs affected (*p* = 0.037).

The results of the ANOVA also showed significantly lower TSH during hospitalization in patients with larger post-COVID-19 lung involvement and damage noted on post-COVID-19 MSCT; *p* = 0.028 for the whole model. Significant differences were found between the patients with no lung involvement, those with lung involvement up to 30%, and those with more than 30% of lung involvement (*p* values represent post hoc Fisher LSD test), Figure 4. 

## 4. Discussion

The interaction between SARS-CoV-2 and thyroid function is bidirectional and complex, and it is not yet fully understood. There is no clear evidence that patients with existing thyroid dysfunction are at risk of developing more severe COVID-19 disease [10,11,12,13]. Data related to thyroid disturbances during COVID-19 in individuals without pre-existing thyroid disorders are even more inconclusive. Therefore, this study contributes to the understanding of TSH as a potential prognostic marker for COVID-19 patients. 

In our study, lower/suppressed TSH level during hospitalization for COVID-19 was associated with worse short-term outcomes, including admission to the ICU, and the need for higher oxygen supplementation via HFOT, or mechanical ventilation. Moreover, patients with lower/suppressed TSH more often died. On the other hand, higher TSH values correlated with better blood oxygen saturation and tended to be more frequent findings among COVID-19 survivors. Non-thyroidal illness (NTI) is a well-known phenomenon reported in critically ill patients in many diseases. It is characterized by decreased free triiodothyronine (fT3) levels and low or normal TSH and high, normal, or low fT4 levels [14,15]. One of the shortcomings of this study was that fT3 and fT4 were not assessed routinely during hospitalization, owing to the retrospective nature of the study, so we could not differentiate patients with NTI and hyperthyroidism. Furthermore, TSH was tested while most patients were treated with glucocorticoids, an additional factor affecting the pituitary–thyroid axis. Therefore, it is difficult to exclude the effect of corticosteroids and systemic inflammation on the pituitary–thyroid axis, leading to a possible misdiagnosis of thyroid dysfunction in severe cases of COVID-19. Nevertheless, our results suggest significantly lower TSH levels in female patients on corticosteroid therapy, but it did not affect their survival. NTI is considered the most frequent thyroid-related problem in COVID-19 care, particularly in hospitalized patients and in intensive care units [10]. In a retrospective study by Chen M et al., patients hospitalized for COVID-19 infection had significantly lower values of TSH than normal compared with healthy controls and non-COVID-19 pneumonia patients with a similar degree of severity. After recovery, the levels of all thyroid hormones returned to the normal value, with no significant differences in thyroid function [16]. In our patients, we did not see a strong association between the laboratory parameters associated with the severity of viral disease (such as IL-6 and D-dimer) with TSH. However, CRP and procalcitonin did show a weak correlation with TSH, which might be cautiously interpreted as the relation of TSH and disease severity. Khoo et al. aimed to detail the acute effects of COVID-19 on thyroid function. In their study of 50 patients, significant differences in TSH were found comparing baseline vs. admission and admission vs. follow-up values. On the contrary, there was no significant difference between baseline vs. follow-up values [17]. In our patient cohort, we observed the rise in TSH levels in 75% of cases during the post-COVID-19 follow-up. Whether this can be a possible explanation for the non-specific complaints some patients have during the post-COVID-19 follow-up is debatable. Low TSH during hospitalization was, besides being a predictor of worse short-term outcomes, also a significant predictor for persistent lung involvement measured by chest MSCT 6 to 8 weeks during post-COVID, whereas we found no correlations between post-COVID-19 TSH and post-COVID-19 lung involvement.

On the other hand, post-mortem studies suggest morphological and pathological changes in endocrine glands, including the thyroid, in people affected by the coronavirus family not previously diagnosed with thyroid disease [18,19]. In the study of Muller et al. patients treated in high-intensity care units (HICU) due to COVID-19 showed lower TSH levels than SARS-CoV-2-negative patients admitted to the same HICU. Furthermore, serum fT4 levels showed no difference between the groups, while the fT3 levels were low in both groups [12]. In our patient cohort, we only had fT4 levels determined for 72 patients, as a secondary level analysis in case of abnormal TSH findings, and in 56.9% of cases it was normal, while slightly elevated fT4 was detected in 41.7% of patients ). Unfortunately, fT3 was not determined. Therefore, due to the small sample size and the absence of fT3, we cannot interpret the findings and differentiate between NTI syndrome and hyperthyroidism due to other causes. 

The cases of subacute thyroiditis with a possibility of clinically manifest hyperthyroidism, as well as the cases of autoimmune thyroiditis or Graves’ disease triggered by the “cytokine storm” induced by SARS-CoV-2 infection, have been described so far [20,21,22,23]. As mentioned earlier, we did not detect the correlation between cytokine levels (specifically IL-6) and TSH levels. 

There is limited knowledge of thyroid function in patients who have recovered from COVID-19 and the medical literature lacks data about the potential thyroid influence on non-specific symptoms often described during the post-COVID-19 period, which negatively influences patients’ quality of life and recovery. Due to the retrospective nature of the study, we could not investigate non-specific symptoms that patients reported. Therefore, we aimed to investigate whether there is an association between objective changes in lung parenchyma (seen on chest MSCT after hospitalization) and serum TSH. It is known that the T3 receptor is present in the lung in the alveolar type II cells that are believed to be involved in recovery after lung injury [24]. Furthermore, studies in rats showed that hypothyroid rats are less able to clear fluid from their lungs, and fluid clearance can be ameliorated by liothyronine administration [25,26]. In our study, those patients who had lower TSH during hospitalization also had worse middle-term outcomes and more pulmonary parenchyma affected by COVID-19. It would be interesting to see whether those are the patients that had low fT3. In the study conducted by Ahn J. and colleagues on 119 Korean patients hospitalized due to COVID-19 infection, TSH and T3 levels were significantly lower in patients who died from COVID-19, as well as in those with severe and critical forms of the disease, compared to those with milder forms. Additionally, patients in the lowest tertile of TSH were more frequently mechanically ventilated than those in the middle or highest tertile of TSH levels. While the degree of reduction in TSH and T3 levels demonstrated a significant correlation with the severity of COVID-19, low T3 levels were identified as having prognostic significance [27]. Pappa and colleagues, in their study on 128 Greek patients, also demonstrated an association between low serum TSH levels and adverse outcomes in hospitalized COVID-19 patients. The authors therefore suggested integrating TSH levels into multivariable machine learning classification algorithms, as it shows promise for outcome prediction with high accuracy [28]. The study by Guven suggested fT3 as a potential predictor of the severity of COVID-19 [29]. The same conclusion was also reached by Dabas and colleagues who found a connection between low fT3 and elevated IL-6 (*p* = 0.021) and death risk (*p* = 0.031) in COVID-19 patients but no association on the other hand between TSH and COVID-19 outcomes [30]. Similar findings were reported by Dincer Yazan C. and collaborators in their work on 205 patients with COVID-19 in Turkey, demonstrating that patients with lower TSH and fT3 levels were more often admitted to the ICU and had a higher mortality rate from COVID-19 compared to euthyroid patients [31]. Currently, a phase 2 clinical trial (NCT 04115514) is being conducted that is investigating liothyronine as a treatment for ARDS in humans, including that associated with COVID-19 [32]. 

One of the main shortcomings of this study, owing to the retrospective analysis, was that fT3 and fT4 were not assessed during the hospitalization. In addition, the serum TSH was tested while patients were receiving glucocorticoids. Furthermore, the patients included in this study were hospitalized patients with moderate–severe disease. Thus, we lack data from patients with milder disease forms. In addition, chest MSCT was not available both during hospitalization and in the post-COVID-19 follow-up for all patients. Nevertheless, to our knowledge, this is the first study investigating the serum TSH and the COVID-19-associated outcomes of patients in a population drawn from the first and largest COVID-19 hospital in Croatia, with a follow-up to assess post-COVID-19 pulmonary involvement. The results of our research may indicate that lower TSH might be a predictor of worse immediate and middle-term outcomes.

Other considerations and limitations requiring clinicians’ attention are related to high sensitivity and low specificity associated with the cut-off value of TSH 0.5 mIU/L. The set specific threshold exhibits a predisposition toward yielding positives and false positives, emphasizing the importance of identifying patients at risk and the potential need for mechanical ventilation. Lower specificity implies a compromised ability of the test to accurately discern patients devoid of risk. However, in our opinion, designating a patient as at risk and providing additional observation is probably less harmful as opposed to the converse scenario. It is imperative to emphasize that TSH provides only supplementary laboratory aid, which should be utilized with caution and only in an adjunct to established standard procedures employed for evaluating the severity of the condition. As there is no current gold-standard test identifying COVID-19 patients at risk for worse outcomes at an early stage of the disease, any additional cheap and easily available tool, such as TSH, might be useful.

## 5. Conclusions

In conclusion, although sick euthyroidism is the most frequent thyroid-related problem encountered in COVID-19 patients, especially those with more severe disease, thyrotoxicosis and hypothyroidism have also been reported. Physicians should be aware of possible connections between SARS-CoV-2 and the thyroid. Further prospective studies including fT3 and fT4 are needed to investigate the impact of COVID-19 on the hypothalamic–pituitary–thyroid axis and vice versa, as well as the impact of thyroid changes on outcomes of patients with COVID-19. An isolated measurement of TSH, according to our results, in case of lower/suppressed levels, can be a useful marker of worse short- and middle-term outcomes and might help in additionally detecting patient cohorts in need of more thorough and prolonged follow-up.

## Figures and Tables

**Table 1:** Patients’ characteristics, hospital stay, and laboratory findings.

	Valid N	Mean	Median	Lower Quartile	Upper Quartile	Std.Dev.
Age (years)	580	66.93	68.00	59.00	78.00	13.31
Length of hospitalization (days)	580	16.06	13.00	8.00	21.00	10.89
SpO_2_ at admission	580	88.22	90.00	85.00	95.00	9.78
Body temperature at admission (°C)	580	37.71	37.80	36.50	38.70	1.18
BMI (kg/m^2^)	382	29.39	28.94	25.53	32.49	5.84
TSH (during hospitalization) (mIU/L)	580	1.16	0.790	0.38	1.62	1.08
TSH (post-COVID) (mIU/L)	207	1.99	1.680	1.15	2.41	1.51
IL-6 (pg/mL)	172	188.92	63.83	21.60	167.86	334.20
CRP (mg/L)	580	104.66	86.35	39.15	153.35	84.97
D-Dimer (mg/L)	436	1.72	1.12	0.60	2.50	1.45
PCT (ng/mL)	361	1.49	0.18	0.09	0.48	7.30

BMI—body mass index; IL-6—interleukin-6; SpO_2_—blood oxygen saturation; CRP—C reactive protein; PCT—procalcitonin.

**Table 2:** Differences in TSH levels during the hospitalization between patients with better or worse outcomes (results of Mann–Whitney U test, as homogeneity of variance between the groups was violated).

	Rank Sum SURVIVED N = 419	Rank Sum DIED N = 161	U	Z	*p*-Value	Z Adjusted	*p*-Value
TSH (mIU/L) hospitalization	126,719	41,771	28,730	2.7661	0.0057	2.7662	0.0057
	No ICU N = 398	ICU N = 182					
TSH (mIU/L) hospitalization	122,284.5	46,205.5	29,552.5	3.5590	0.0004	3.5591	0.0004
	Mech. Vent. N = 453	Mech. Vent. N = 127					
TSH (mIU/L) hospitalization	138,157.0	30,333.0	22,205.0	3.9306	0.0001	3.9307	0.0001

**Table 3:** Corticosteroid use with respect to TSH levels among male and female COVID-19 patients.

TSH (mIU/L) Hospitalization	Rank Sum No Cortico	Rank Sum Cortico	U	Z	*p*-Value	Z Adjusted	*p*-Value
TOTAL	28,746	127,774	16,618	2.9520	0.0032	2.9520	0.0032
MEN	4872.0	37,614.0	3684.0	0.7802	0.4353	0.7802	0.4353
WOMEN	9101.5	26,945	4578.5	2.7627	0.0057	2.7628	0.0057

**Table 4:** TSH changes in the post-COVID-19 period (results of Student-*t* test for dependent samples for total sample and according to sex; male = 129 and female = 78).

TOTAL	Mean	Std.Dev.	Diff.	Std.Dev. Diff.	t	df	*p*
TSH (mIU/L) hospitalization	1.146	1.006					
TSH (mIU/L) post-COVID-19	1.987	1.510	−0.841	1.599	−7.57	206	0.000
MALE							
TSH (mIU/L) hospitalization	1.180	1.211					
TSH (mIU/L) post-COVID-19	1.897	1.459	−0.717	1.591	−5.12	128	0.000
FEMALE							
TSH (mIU/L) hospitalization	1.726	3.054					
TSH (mIU/L) post-COVID	2.723	4.568	−0.998	2.555	−3.56	82	0.001

## Data Availability

The data that support the findings of this study are available from the corresponding author (I.B.-C.), upon reasonable request.
